# Regeneration of Stevia Plant Through Callus Culture

**DOI:** 10.4103/0250-474X.51954

**Published:** 2009

**Authors:** R. M. Patel, R. R. Shah

**Affiliations:** Plant Tissue Culture Laboratory, ASPEE College of Horticulture and Forestry, Navsari Agricultural University, Navsari-396 450, India

**Keywords:** Stevia, regeneration, glucose, glycoside, explant, callus, hardening

## Abstract

*Stevia rebaudiana* Bertoni that conventionally propagated by seed or by cuttings or clump division which has a limitation of quality and quantity seed material. In present study, callus culture technique was tried to achieve rapid plant multiplication for quality seed material. Callus induction and multiplication medium was standardized from nodal as well as leaf sagments. It is possible to maintain callus on Murashige and Skoog medium supplemented with 6-benzyl amino purine and naphthalene acetic acid. Maximum callus induction was obtained on Murashige and Skoog medium incorporated with 6-benzyl amino purine (2.0-3.0 mg/l) and naphthalene acetic acid (2.0 mg/l) treatments. However, Murashige and Skoog medium containing 2.0 mg/l 6-benzyl amino purine+2.0 mg/l naphthalene acetic acid was found to be the best for callus induction. Higher regeneration frequency was noticed with Murashige and Skoog medium supplemented with 2.0 mg/l 6-benzyl amino purine+0.2 mg/l naphthalene acetic acid. Regenerated plants were rooted better on ¼ Murashige and Skoog strength supplemented with 0.1 mg/l indole-3-butyric acid. The rooted plantlets were hardened successfully in tera care medium with 63 per cent survival rate. The developed protocol can be utilized for mass production of true to type planting material on large scale independent of season, i.e. external environmental conditions.

*Stevia rebaudiana* Bertoni belongs to Asteraceae family is perennial herb native to Paraguay and Southern Brazil. The leaves of the plant contain glycosides, which have chemical and pharmacological characteristics suitable for use in human diet as a natural calory-free agent. Diterene glucosides is 100-400 times more sweeter than glucose[1]. The eight types of glycosides are rebaudioside A, rebaudioside B, rebaudioside C (dulcoside A), rebaudioside D, rebaudioside E, rebaudioside F, steviolbioside A and dulcoside A are identified. On commercial scale, utilization of these sweets are in vouge since the early seventies in Japan. Recently, it's liberation for human use in Brazil has made importance of Stevia cultivation, commercially. Stevia making strides as the best alternative of the table sugar in the years to come[2]. Stevia is cultivated in several countries in world viz. Japan, Brazil, South East Asia, China and Canada[3]. The plant is propagated by seed or by cutting. Althogh seed propagation is very common method, seed is not efficient becauce of low fertility[4] and self incompatibility of the flowers[5]. Whlie propagation through cutting has a limitation on account of the low number of new plants, simultaneusly, from a donor plant. Stevia can be grown throughout India in subtroprical climatic region. Therefore, considering its scope and future need of planting material, this experiment was conducted to standardise protocol for rapid multiplication of Stevia through callus culture method.

Nodal segments and leaves were collected as source of explants from Stevia plant. Nodal segments of about 1.5 cm length and leaves were isolated from collected explants. The explants were washed in running tap water for about 30 min and then, treated with 10 per cent solution of detergent for 5 min. The traces of detergent were removed by wasing thoroughly with double glass distilled water. The explants were then, surface sterilized by using 0.1% mercuric chloride solution for 3 minutes under aseptic conditions in a laminar air-flow cabinet followed by rinsing four times with sterilized double glass distilled water. The size of sterilized nodes were further reduced to 0.8 cm by trimming both the ends. While in case of leaf explants, sterilized leaves were cut into 0.5 cm^2^ size pieces for inoculation on the medium.

The prepared explants were inoculated on nutrient Murashige and Skoog (MS) medium[6] supplemented with 6-benzyl amino purine (BAP- 1, 2 and 3 mg/l) in combination with naphthalene acetic acid (NAA- 0.2 and 2.0 mg/l). The cultures were inoculated at 26±2° temperature in air-conditioned culture room and provided with 1000 lux light intensity from fluorescence cool tube light. The callus obtained on different treatments were recultured to S_3_ (MS supplemented with 2.0 mg/l BAP and 0.2 mg/l NAA) for regenerarion of shoots. The shoots obtained on regeneration medium were excised and transferred to rooting medium for *in vitro* rooting. MS liquid medium with quarter and half strength were tried. Each medium was supplemented with different levels of indole-3-butyric acid (IBA levels: 0.05, 0.1, 0.2, 0.5, and 1.0 mg/l). Observations on establishment and proliferation were recorded after three and four weeks of incubation of cultures, respectively, while in case of rooting, it was recorded after three weeks.

The rooted plantlets were transferrred in polyethylene bags filled with four types of potting mixtures i.e. tera care; soil:tera care; soil:leaf mould and soil:leaf mould:tera care each in equal proportions. Survival % of plantlets were recorded after three weeks.

The data on culture establishment of both types of explants i.e. leaf piece and nodal segment on each treatment are presented in Table 1. Among both the explants, establishment of nodal segments was found significantly superior than leaf pieces. The establishment of explants was statistically infuenced by treatments. The maximum establishment was recorded on MS medium supplemented with 1.0 mg/l BAP+0.2 mg/l NAA treatment. Nodal segments established 100% on MS medium supplemented with 1.0-2.0 mg/l BAP+0.2-2.0 mg/l NAA. While, piece of leaf explants, showed better establishment (58.58%) on S_2_ (MS supplemented with 1.0 mg/l BAP+2.0 mg/l NAA) medium than other treatments.

**TABLE 1:** EFFECT OF PLANT GROWTH REGULATORS ON RESPONSE OF EXPLANT SOURCES TO CALLUS STATUS

Treatment	BAP (mg/l)	NAA (mg/l)	Establishment (%)*	Callusing induction (%)	Intensity	Callus colour	No. shoots/ Explant	Length shoots/ Explant
E-Explant								
E_1_- Nodal segment			95.0 (83.187)	95.00				
E_2_- Leaf piece			53.3 (47.008)	53.33				
S-Treatment								
S_1_	1.0	0.2	80.0 (69.934)	80.00				
S_2_	1.0	2.0	86.2 (73.820)	86.25				
S_3_	2.0	0.2	71.2 (64.826)	71.25				
S_4_	2.0	2.0	80.0 (69.934)	80.00				
S_5_	3.0	0.2	63.7 (56.972)	63.75				
S_6_	3.0	2.0	63.7 (55.098)	63.75				
E × S Interaction								
E_1_- Nodal segment								
S_1_	1.0	0.2	100 (89.058)	100.00	+	Whitish Green	2.05	1.050
S_2_	1.0	2.0	100 (89.058)	100.00	++	White green	0.95	0.600
S_3_	2.0	0.2	100 (89.058)	100.00	+	Green	3.05	0.800
S_4_	2.0	2.0	100 (89.058)	100.00	+	Light Green	0.80	0.550
S_5_	3.0	0.2	90.0 (76.233)	90.00	+	Light Green	2.00	0.700
S_6_	3.0	2.0	80.0 (66.656)	80.00	+		0.75	0.500
E_2_- Leaf piece								
S_1_	1.0	0.2	60.0 (50.811)	60.00	+	Whitish Green	0.0	0.0
S_2_	1.0	2.0	72.5 (58.583)	72.50	++	Whitish Green	0.0	0.0
S_3_	2.0	0.2	42.5 (40.594)	42.50	+	Whitish Green	0.0	0.0
S_4_	2.0	2.0	60.0 (50.811)	60.00	+	Whitish Green	0.0	0.0
S_5_	3.0	0.2	37.5 (37.711)	37.50	+	Brown Green	0.0	0.0
S_6_	3.0	2.0	47.5 (43.540)	47.50	+	Brown Green	0.0	0.0
E-Explant								
S. Em. ±			1.482					
CD at 5%			4.253					
S-Treatment								
S. Em. ±			4.567				0.113	0.072
CD at 5%			7.367				0335	0.215
E × S Interaction								
S. Em.±			3.630					
CD at 5%			10.418					
CV %			11.15				14.13	20.76

* Arc sign transformed values are presented in parentheses. E= Explant source; S = Treatment, Medium : MS, Incubation: 3 weeks

It is apparently seen from the data (Table 1) that in both types of explants, callus was induced on all the treatments. However, S_2_ (MS supplemented with 1.0 mg/l BAP+2.0 mg/l NAA) induced better callus among all the treatments. The colour of callus ranged from green to white green to brown yellow in different treatments. At the end of 1st subculture, the intensity of callus was increased and maximum callus was noticed on S_2_ treatment in both the explants (Table 2). However, higher intensity was observed in nodal segments than leaf pieces. Normally, nodal segments produced globular callus.

**TABLE 2:** GROWTH AND TYPE OF CALLUS AFTER FIRST SUBCULTURE AS INFLUENCED BY BAP, NAA AND EXPLANT SOURCES

	Treatment		Fresh weight of callus (g)	Colour of Callus	Callus status
	
	BAP (mg/l)	NAA (mg/l)			
E1- Nodal segment					
S_1_	1.0	0.2	1.60	Yellow Green	Globular
S_2_	1.0	2.0	1.82	Yellow Green	_
S_3_	2.0	0.2	1.52	Yellow Green	Globular
S_4_	2.0	2.0	1.70	Yellow Green	Globular
S_5_	3.0	0.2	1.75	Yellow Green	Smooth
S_6_	3.0	2.0	1.52	Brown Yellow	Globular
E2- Leaf piece					
S_1_	1.0	0.2	1.75	Light Green	Smooth
S_2_	1.0	2.0	2.01	Green	Subglobular
S_3_	2.0	0.2	1.60	Light Green	Smooth
S_4_	2.0	2.0	1.95	Green	Subglobular
S_5_	3.0	0.2	1.92	Brown Yellow	Smooth
S_6_	3.0	2.0	1.88	Brown Yellow	Subglobular

E = Explant source; S = Treatment, Medium: MS, Incubation: 4 weeks

The treatment S_3_ (MS supplemented with 2.0 mg/l BAP+0.2 mg/l NAA) registered maximum regeneration of shoots. (Table 1). Therefore, calli developed on other media combinations were also transferred to S_3_ treatment for regeneration of shoots from callus. The shoots were regenerated through organogenesis from callus. The regeneration frequency was significantly lower in case of S_1_ and S_6_ calli. Maximum number of shoots were regenerated, when callus produced on S_2_ treatment was transferred to S_3_ treatment, whereas, length of shoot was noticed significantly higher on S_1_ followed by S_2_ and S_3_ (Table 3).

**TABLE 3:** REGENERATION POTENTIAL OF STEVIA CALLUS ON MEDIUM SUPPLEMENTED WITH BAP AND NAA

Callus obtained on treatment	Callus tranferred to treatment	Regeneration frequency (%)	No. of shoot /Callus	Average shoot length (cm)
S_1_ (1.0 mg/l BAP + 0.2 mg/l NAA)	S_3_	90.0	2.250	4.450
S_2_ (1.0 mg/l BAP + 2.0 mg/l NAA)	S_3_	100.0	4.650	3.450
S_3_ (2.0 mg/l BAP + 0.2 mg/l NAA)	S_3_	100.0	3.400	2.050
S_4_ (2.0 mg/l BAP + 2.0 mg/l NAA)	S_3_	100.0	2.550	1.500
S_5_ (3.0 mg/l BAP + 0.2 mg/l NAA)	S_3_	100.0	2.400	1.650
S_6_ (3.0 mg/l BAP + 2.0 mg/l NAA)	S_3_	80.0	2.450	1.600
S.Em.±			0.108	0.081
CD at 5%			0.320	0.242
CV %			7.32	6.67

Medium: MS, Incubation : 4 weeks, S = Treatment

The data on rooting response to different levels of IBA supplemented in half strength MS and one fourth strength MS medium are presented in Table 4. It was noticed that rooting response was statistically not influenced by the strength of MS medium and concentrations of IBA. However, interaction effect of strength of MS medium and concentrations of IBA was found significant. One hundred per cent rooting in the cultures was recorded on half strength MS medium supplemented with 0.2-1.0 mg/l IBA, whereas in case of quarter strength medium, it was noticed 100% when supplemented with 0.05-0.5 mg/l IBA. Maximum number of roots (9.47 roots/plantlet) were recorded on ¼ MS strength medium supplemented with 0.1 mg/l IBA treatment among all the treatments. Length of root was significantly infuenced by strength of MS medium and concentrations of IBA. In case of treatment combinations, maximum length of root was registered on quarter strength MS medium supplemented with 0.2 mg/l IBA treatment.

**TABLE 4:** EFFECT OF MEDIUM STRENGTH AND IBA CONCENTRATION ON *IN VITRO* ROOTING OF STEVIA SHOOTS

Treatment	Rooting (%)*	No of roots/shoot	Average length of root (cm)
Strength of MS medium (S)			
S_1_-Half strength MS medium	94.66 (83.084)	6.666	2.226
S_2_-Quarter strength MS medium	97.33 (85.638)	6.853	3.100
IBA concentration (R)			
R_1_- 0.05 mg/l IBA	90.00 (78.398)	7.600	1.466
R_2_- 0.1 mg/l IBA	96.66 (84.783)	8.666	3.200
R_3_- 0.2 mg/l IBA	100.00 (89.058)	6.400	3.266
R_4_- 0.5 mg/l IBA	100.00 (89.058)	5.833	3.166
R_5_- 1.0 mg/l IBA	93.34 (80.508)	5.300	2.216
SXR Interaction			
S_1_-Half strength MS medium			
R_1_- 0.0 5 mg/l IBA	80.00(67.738)	8.266	1.466
R_2_- 0.1 mg/l IBA	93.33(80.508)	7.866	3.200
R_3_- 0.2 mg/l IBA	100.00 (89.058)	6.133	2.666
R_4_- 0.5 mg/l IBA	100.00 (89.058)	5.400	2.600
R_5_- 1.0 mg/l IBA	100.00 (89.058)	5.666	1.200
S_2_-Quarter strength MS medium			
R_1_- 0.05 mg/l IBA	100.00 (89.058)	6.933	1.466
R_2_- 0.1 mg/l IBA	100.00 (89.058)	9.466	3.200
R_3_- 0.2 mg/l IBA	100.00 (89.058)	6.666	3.866
R_4_- 0.5 mg/l IBA	100.00 (89.058)	6.266	3.733
R_5_- 1.0 mg/l IBA	86.67 (71.959)	4.933	3.233
Strength of MS medium (S)			
S.Em.±	2.337	0.143	0.090
C.D. at 5%	NS	NS	0.267
IBA concentration (R)			
S.Em.±	3.695	0.226	0.143
C.D. at 5%	NS	0.668	0.422
SXR Interaction			
S. Em.±	5.226	0.320	0.202
CD at 5%	15.418	0.945	0.597
CV %	10.73	8.21	13.17

* Arc sign transformed values are presented in parentheses.S = Medium strength (S_1_ = Half strength; S_2_ = Quarter strength) R = IBA concentration (R_1_=0.05 mg/l; R_2_=0.1 mg/l; R_3_=0.2 mg/l; R_4_=0.5 mg/l and R_5_=1.0 mg/l). Incubation: 3 weeks

The survival rate of plantlets was infuenced by potting mixtures. Maximum number of survival of plantlets (63 per cent) was found in tera care potting medium (Table 5). Addition of leaf mould or soil in tera care reduced the survival rate of plantlets.

**TABLE 5:** EFFECT OF POTTING MIXTURE ON SURVIVAL OF PLANTLETS DURING HARDENING

Treatment	Survival of plantlet (%)
1. Tera care	63.0
2. Soil+Tera care	47.0
3. Soil+Leaf mould	34.0
4. Soil+Leaf mould+Tera care	40.0

Duration: 3 weeks

For the culture of a number of callus tissues, auxin is the essential supplement which is needed to be added to the basal medium supplying inorganic ions and sugars[7]. Navarro *et al*.[8] also noted that auxin was essential supplement for induction of callus. The addition of cytokinin in the auxin medium has an additive effect on tissue growth has been demonstrated by other workers in a number of plant species[6]. Incorporation of BAP was also found to have beneficial effect on growth of callus in the present investigation.

The role of cytokinin in shoot organogenesis is well established[9–11]. In the present investigation, MS medium supplemented with high ratio of cytokinin (BAP) and auxin (NAA) responsed favourably for regeneration of shoots. However, in other studies of micropropagation[4], maximum number of shoots were reported on MS medium supplemented with 0.6 mg/l BA. The difference may be due to source of explant and hormonal levels used in callus culture.

Addition of Auxin in the MS medium at adequate levels enhanced root formation. In our studies better response of rooting were observed on ¼ strength MS medium supplemented with 0.1 mg/l IBA. The present investigation is in agreement with findings of Ferreira and Handro[12] who also reported optimum concenrtation for root formation at 0.1 mg/l IBA. On the other hand, better rooting response was reported by Tadhani *et al.*[4] on MS medium supplementd with 1 mg/l IBA in case of Stevia. The variation may be due to the source of explant, the change in microclimate and may vary with the strength of rooting MS medium, which was observed in the present study.

*In vitro* plants need to be hardened before being transplanted in the field. In the present study, potting mixture tera care performed well with better survival of the plantlets. The potting mixture used may help in giving better grip for the rooting and ample aeration. The survival of *in vitro* plants also depends upon plant species and the potting mixture used for raising *in vitro* plants under greenhouse conditions. The other reports on potting mixtures such as peat:perlite[13], sand:soil:vermiculite[11], etc. were reported for establishment of *in vitro* plants under greenhouse conditions. The technique developed may be utilized efficiently for producing true type and disease free planting materials on large scale during any time of the year.
